# Impact of preoperative weight loss achieved by gastric balloon on peri- and postoperative outcomes of bariatric surgery in super-obese patients: a retrospective matched-pair analysis

**DOI:** 10.1007/s00423-022-02472-1

**Published:** 2022-03-08

**Authors:** Ilona Hering, Luise Dörries, Sven Flemming, Laura Krietenstein, Ann-Kathrin Koschker, Martin Fassnacht, Christoph-Thomas Germer, Mohammed K. Hankir, Florian Seyfried

**Affiliations:** 1 Department of General, Visceral, Transplantation, Vascular and Pediatric Surgery; Center of Operative Medicine (ZOM), University Hospital of Wuerzburg, Wuerzburg, Germany; 2 Department of Internal Medicine I, Division of Endocrinology and Diabetes, Center of Internal Medicine (ZIM), University Hospital of Wuerzburg, Wuerzburg, Germany

**Keywords:** Obesity, Super-obesity, Intragastric balloon, Sleeve gastrectomy, Roux-en-Y gastric bypass

## Abstract

**Background:**

An intragastric balloon is used to cause weight loss in super-obese patients (BMI > 60 kg/m^2^) prior to bariatric surgery. Whether weight loss from intragastric balloon influences that from bariatric surgery is poorly studied.

**Methods:**

In this retrospective, single-center study, the effects of intragastric balloon in 26 patients (BMI 69.26 ± 6.81) on weight loss after bariatric surgery (primary endpoint), postoperative complications within 30 days, hospital readmission, operation time, and MTL30 (secondary endpoints) were evaluated. Fifty-two matched-pair patients without intragastric balloon prior to bariatric surgery were used as controls.

**Results:**

Intragastric balloon resulted in a weight loss of 17.3 ± 14.1 kg (BMI 5.75 ± 4.66 kg/m^2^) with a nadir after 5 months. Surgical and postoperative outcomes including complications were comparable between both groups. Total weight loss was similar in both groups (29.0% vs. 32.2%, *p* = 0.362). Direct postoperative weight loss was more pronounced in the control group compared to the gastric balloon group (29.16 ± 7.53% vs 23.78 ± 9.89% after 1 year, *p* < 0.05 and 32.13 ± 10.5% vs 22.21 ± 10.9% after 2 years, *p* < 0.05), who experienced an earlier nadir and started to regain weight during the follow-up.

**Conclusion:**

A multi-stage therapeutic approach with gastric balloon prior to bariatric surgery in super-obese patients may be effective to facilitate safe surgery. However, with the gastric balloon, pre-treated patients experienced an attenuated postoperative weight loss with an earlier nadir and earlier body weight regain. This should be considered when choosing the appropriate therapeutic regime and managing patients’ expectations.

## Introduction

The obesity pandemic is one of the most challenging health and socioeconomic problems of our time [1, 2]. This is largely because obesity is closely associated with various debilitating comorbidities such as type 2 diabetes, cardiovascular disease, and nonalcoholic fatty liver disease (NAFLD) as well as psychiatric disorders which all significantly impair quality of life and reduce life expectancy [3].

There is a clear and undisputed body of evidence showing that bariatric surgery is currently the best treatment option to combat obesity as it leads to significant and sustained weight loss, reduction or even remission of obesity-associated comorbidities, improved functional status, and prolonged overall survival [4–6]. It has further been widely shown that bariatric surgery is safe to perform with considerably low rates of perioperative morbidity and mortality in specialized centers [7–9].

Bariatric surgery in super-obese patients (BMI > 60 kg/m^2^) can be technically challenging for the surgeon to perform because of the excessive visceral obesity and enlarged (fatty) liver [10, 11]. Since these patients are also more likely to be diagnosed with more advanced comorbidities including end-organ damage, bariatric surgery is associated with increased perioperative morbidity and mortality [12–14].

A number of therapeutic strategies including more-stage concepts and the use of conservative or interventional preoperative weight loss by intragastric balloon have been developed to safely and successfully treat super-obese, high-risk bariatric surgery patients [1, 15–17]. We have implemented a two-stage approach with intragastric balloon insertion as a first step, followed by its removal after 6 months, before concomitant laparoscopic sleeve gastrectomy (LSG) or Roux-en-Y gastric bypass (RYGB). With the increase of surgical and anesthesiologic experience in this high-risk patient population, along with a FDA warning on possible severe side effects of gastric balloon treatment [18], we changed our in-house policy and performed primary bariatric surgery after a short period of preoperative weight loss from caloric restriction [19] if technically feasible (single-stage approach).

The purpose of this single-center, a matched-pair study was to analyze the peri- and postoperative outcome of a two-stage (intragastric balloon with consecutive bariatric surgery) vs. single-stage approach (bariatric surgery alone) in super-obese patients during a 2-year follow-up.

## Material and methods

### Institution

The bariatric center at the University Hospital of Würzburg is certified as a Center of Reference for bariatric and metabolic surgery from the German Society of General- and Visceral Surgery (DGAV) and performs more than 150 primary and revisional operative procedures per year on average. All patients referred for bariatric surgery are discussed at a multidisciplinary team (MDT) meeting including at least an endocrinologist, psychologist/psychiatrist, nutrition expert, and bariatric surgeon and are treated according to national guidelines.

### Gastric balloon insertion

We used a single spherical silicone-made balloon (Bioenteric Intragastric Balloon, BIB) of about 13 cm in diameter, arriving commercially compressed and impacted at the end of a filling tube attached to a radiopaque self-sealing valve. After an initial diagnostic endoscopy, the balloon placement assembly was inserted orally into the gastric fundus and a volume of 700 mL saline solution was used for balloon inflation through a closed infusion circuit, the whole procedure was performed under direct endoscopic supervision.

### Protocol and study population

In this single-center study, all consecutive patients scheduled for a two-stage strategy (intragastric balloon with consecutive bariatric surgery) were identified from our prospectively collected database (*n* = 30). All data was prospectively collected and transferred to the National Database (StuDoQIMBE). Four patients (13.3%) had to be excluded since no operation was performed after balloon removal. Of these excluded patients, two suffered from severe vomiting precluding continuation of balloon treatment. One patient experienced a balloon dislocation and had to undergo emergency surgery. The fourth excluded patient chose to be treated in another center and was lost to follow-up. Of the remaining 26 patients, intragastric balloon treatment was accompanied by adverse side effects such as vomiting and heartburn in two (7.7%) but did not prompt balloon removal.

In our schedule, balloon removal and bariatric surgery were not performed at the same time in order to reduce potential gastric fundal inflammation and hypertrophy of the gastric wall which have been shown to result in an increased leakage rate from the staple line [20]. Thus, the time between balloon removal and surgery was 21.0 ± 18.8 days. In order to create a control cohort (“control group”) for the remaining 26 patients, 52 patients receiving primary surgery were derived from the same database with the following matching criteria at the time point of surgery: sex, age, BMI, comorbidity, and subsequent surgical procedure. This resulted in a 2:1 matched-pair analysis which has stronger statistical power.

### Outcome

Outcome parameters were treatment results of gastric balloon as well as a direct comparison of the perioperative and 2-year outcome in the “gastric balloon” vs. “control group.” The primary endpoint was weight loss within 2 years following surgery. Secondary endpoints included postoperative complications within 30 days, length of hospital stay, hospital readmission, operation time, and MTL30 (mortality, transfer, length of stay) [21].

### Statistical analysis

Descriptive data are presented as median with standard deviation or total numbers with percentage. Differences in patient characteristics were assessed by chi-square test, Fisher`s exact test, or ANOVA test according to data scale and distribution. A *p*-value of < 0.05 was considered statistically significant. Statistical analysis was performed using the MEDAS statistics program (https://www.medas-info.de/module/medas-auswertung).

## Results

### Patient characteristics

As presented in Table 1, both groups did not show any significant differences regarding age, sex, comorbidities (type 2 diabetes and arterial hypertension) and EOSS score, and type of surgical procedure at the time point of surgery. The BMI at the time point of the first presentation in our outpatient clinic was significantly higher in the gastric balloon group compared to patients receiving primary operation (69.26 kg/m^2^ vs. 64.07 kg/m^2^, *p* < 0.01). However, there were no differences between the two groups at the time of point of bariatric surgery (63.0 vs. 63.0 kg/m^2^, *p* = 0.80).

**Table 1:** Baseline characteristics of patients who underwent gastric ballooning compared to the control group

	Gastric balloon	Control group	*p*-value
Number	26	52	
Age (median ± SD; years)	48.24 ± 10.2	47.87 ± 10.1	0.69
Sex (*n*, %) Female Male	9 (34.6) 17 (65.4)	26 (50.0) 26 (50.0)	0.19
Type 2 Diabetes mellitus (*n*, %) Total Insulin dependent Not insulin dependent	15 (57.7) 5 (19.2) 10 (38.5)	21 (41.2) 9 (17.7) 12 (23.5)	0.33
Arterial hypertension (*n*, %)	25 (96.2)	49 (94.2)	1.00
EOSS Score (*n*, %) 1 2 3 Average EOSS Score (median ± SD)	0 (0) 12 (46.2) 14 (53.8) 2.5 ± 0.51	0 (0) 36 (69.2) 16 (30.8) 2.33 ± 0.47	0.14

### Perioperative and postoperative outcomes

In both groups, a laparoscopic approach (100%) was performed and sleeve gastrectomy was the slightly favored surgical approach (gastric balloon group 61.5% vs. control group 67.3%, *p* = 0.62). A conversion to open surgery was necessary for one patient from each group (3.8% vs. 1.9%). Even if the overall operation time was slightly increased in the gastric balloon group (99.08 ± 32.9 vs. 86.02 ± 32.4 min; *p* = 0.10), subgroup analysis for each operation procedure (sleeve gastrectomy and gastric bypass) did not reveal any differences between both groups (Table 2).

**Table 2:** Perioperative data of patients with the gastric balloon and the control group

	Gastric balloon	Control group	*p*-value
Primary bariatric procedure (*n*, %) RYGB LSG	10 (38.5) 16 (61.5)	17 (32.7) 35 (67.3)	0.61
Surgical technique (*n*, %) Laparoscopic Conversion to open	25 (96.2) 1 (3.8)	51 (98.1) 2 (1.9)	0.28
Operation time (median ± SD; min)	99.08 ± 32.9	86.02 ± 32.4	0.10
Operation time RYGB (median ± SD; min)	128.33 ± 29.9	107.12 ± 43.2	0.20
Operation time SG (median ± SD; min)	82.63 ± 21.6	75.47 ± 18.5	0.27
Time of hospital stay (median ± SD; days)	7.15 ± 2.33	7.67 ± 2.72	0.21
Postoperative complications (Clavien-Dindo 3b-4b; *n*, %)	1 (3.8)	1 (1.9)	0.38
Mortality (within 30 days; *n*, %)	0 (0)	0 (0)	1.00
MTL30 positive	0 (0)	0 (0)	1.00
Hospital readmission (within 30 days) (*n*, %)	2 (7.6)	3 (5.7)	1.00

Postoperative outcomes also show comparable results in both groups without relevant disparities (Table 2). There were no differences in postoperative severe morbidity measured by Clavien-Dindo (3a-5) classification (3.8% vs. 1.9%; *p* = 0.38) and hospital readmission (7.6% vs. 5.7%; p = 1.00). Based on these results in addition to no mortality, the MTL30 score was negative for all patients in both groups.

### Weight loss during intragastric balloon and postoperative follow-up

**Fig. 1:**
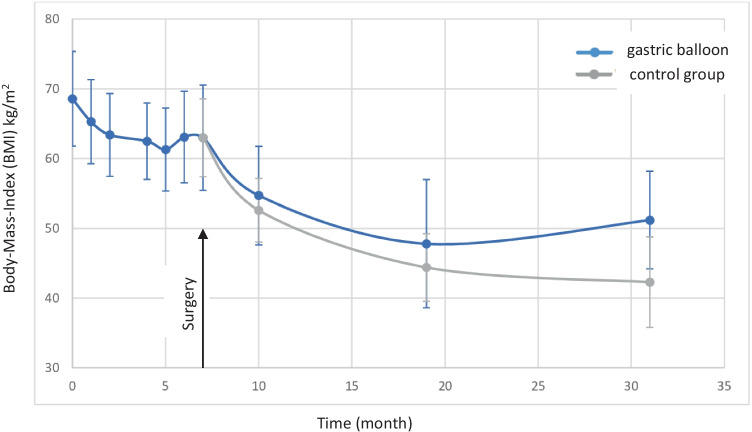
Development of BMI in gastric balloon and control group until 2 years after surgery

During the time period of intragastric balloon treatment (168.1 ± 43.1 days), patients experienced a weight loss of 17.3 ± 14.1 kg (BMI 5.75 ± 4.66 kg/m^2^) (Table 3). Most of the patients with intragastric balloon showed a nadir of weight loss after 5 months with slight weight regain during the further course of treatment (Fig. 1).

**Table 3:** Development of body mass index, excess weight loss, and total weight loss after primary bariatric surgery in patients with gastric ballooning compared to the control group

	Gastric balloon	control group	p-value
Body-Mass-Index (median ± SD; kg/m^2^) Before gastric ballooning/first presentation Prior bariatric surgery 3 months after bariatric surgery 1 year after bariatric surgery 2 years after bariatric surgery	69.26 ± 6.81 63.0 ± 7.55 54.72 ± 7.06 47.80 ± 9.18 51.15 ± 6.99	64.07 ± 5.09 63.0 ± 5.09 52.61 ± 4.55 44.44 ± 4.85 42.25 ± 6.62	< 0.01 0.80 0.10 0.22 < 0.01
EWL (excess weight loss) (median ± SD, %) 1 year after bariatric surgery 2 years after bariatric surgery	40.38 ± 17.5 35.43 ± 16.20	48.51 ± 12.3 53.90 ± 17.9	< 0.05 < 0.05
Total weight loss (median ± SD, %) 1 year after bariatric surgery 2 years after bariatric surgery 1st bariatric intervention to 2 years after Bariatric surgery	23.78 ± 9.89 22.21 ± 10.9 29.0 ± 8.45	29.16 ± 7.53 32.23 ± 10.5 32.2 ± 10.51	< 0.05 < 0.05 0.362

Weight loss was more pronounced in the control group and was significantly different compared to the gastric balloon group as shown in Table 3 (total weight loss: 23.8% vs. 29.2% after 1 year, *p* < 0.05; 22.2% vs. 32.2% after 2 years, *p* < 0.05). Furthermore, patients from the gastric balloon group experienced an early nadir and started to regain weight within the 2 years of follow-up (BMI 47.8 ± 9.18 after 1 year vs. 51.15 ± 6.99 after 2 years) (Fig. 1). Nevertheless, the total weight loss caused by preconditioning due to gastric balloon followed by bariatric surgery was 29.0% and therefore not significantly different to the control group (32.2%, *p* = 0.362) (Table 3). Due to visceral obesity and limited trocar maneuverability, some of the planned RYGB needed to be changed to a sleeve gastrectomy. Analysis of BMI depending on surgical procedure and pre-treatment showed that RYGB resulted in a significant weight loss 2 years after bariatric surgery compared to the control group with sleeve gastrectomy (Table 4). In the gastric balloon group, the weight loss was also more pronounced in the RYGB group. However, significance has been not reached probably due to a small number of patients.

**Table 4:** Development of body mass index depending on pre-treatment (gastric balloon vs. control group) and surgical procedure (RYGB vs. LSG)

	Gastric balloon + LSG (*n* = 16)	Gastric balloon + RYGB (*n* = 10)	*p*-value	Control group + LSG (*n* = 35)	Control group + RYGB (*n* = 17)	*p*-value
Body mass index (median ± SD; kg/m^2^)						
Before gastric ballooning/first presentation	70.1 ± 5.08	68.8 ± 8.69	*p* = 0.646	63.9 ± 5.29	64.3 ± 4.45	*p* = 0.784
Prior bariatric surgery	63.4 ± 4.95	62.4 ± 10.1	*p* = 0.738	63.2 ± 5.86	62.5 ± 4.72	*p* = 0.645
3 months after bariatric surgery	55.6 ± 5.54	53.5 ± 8.19	*p* = 0.471	53 ± 4.47	51.9 ± 4.48	*p* = 0.437
1 year after bariatric surgery	49.9 ± 8.92	44.8 ± 8.02	*p* = 0.246	45.1 ± 3.91	43.3 ± 5.86	*p* = 0.245
2 years after bariatric surgery	51.9 ± 7.98	49.7 ± 3.93	*p* = 0.619	44.1 ± 5.39	38.7 ± 6.93	*p* = 0.038

## Discussion

It has been widely shown that bariatric surgery is a highly effective and safe treatment option to induce sustained and relevant weight loss in severely obese patients accompanied by improvement in quality of life and in increased life expectancy [4, 6]. However, the perioperative handling of super-obese patients (BMI > 60 kg/m^2^) remains challenging and the likelihood of sufficient therapeutic success is less certain [22, 23]. It has also been shown that the perioperative risk in these patients is increased [10]. Thus, concepts that facilitate preoperative weight loss in order to ensure safe technical operability and improve patients’ functional status and associated comorbidity are much needed [24]. Gastric balloon insertion has been used for preconditioning before performing bariatric surgery in super-obese patients, with the aim of significantly reducing visceral fat tissue, liver size, and, thus, improving technical operability [16, 17, 20].

Our study demonstrates that bariatric surgery was technically feasible in all patients who completed the intended 6 months gastric balloon treatment. This could be achieved in 86.7% of all cases (26 of 30 patients). The overall morbidity in our gastric balloon-treated cohort was, however, considerable (17.2%), but in line with previous studies [25, 26]. One patient had to undergo emergency surgery due to balloon dislocation and four patients suffered from severe vomiting and heartburn, necessitating balloon removal in two patients.

Of note, the perioperative morbidity was comparable between the gastric balloon and the control group. Insertion of a gastric balloon often leads to gastric fundal inflammation and hypertrophy of the gastric wall resulting in increased leakage from the staple line and higher perioperative morbidity [16, 20]. There are several possible explanations for why this was not the case in the present study. One reason could be that we chose a sufficient time interval (21 days on average) between gastric balloon removal and bariatric surgery thus allowing resolution of gastric inflammation and intestinal wound healing. Non-surgical adverse events after operations were also not increased in the gastric balloon group since both groups had comparable BMI and comorbidities. Additionally, evidence-based and structured postoperative pathways for obese patients were implemented to reduce postoperative morbidity.

There are reports of increased operation times after balloon insertion. For example, one randomized multi-center trial study showed that operation times for laparoscopic RYGB significantly increased from 174.8 ± 83.1 min to 188.1 ± 98.1 min) after balloon pre-treatment [17]. Even though patients in our cohort were considerably more obese (average BMI at the time point at surgery 63 kg/m^2^ vs. 51 kg/m^2^), operation times for RYGB were shorter. Nevertheless, the increase in operation time after balloon insertion (107.12 ± 43.2 vs. 128.33 ± 29.9) was comparable, although it did not attain statistical significance. The length of hospital stay was not different in both groups compared to other studies [15, 17].

Preconditioning with insertion of an intragastric balloon reduced BMI by 5.8 kg/m^2^ similar to previous studies [16, 17, 26, 27]. Notably, weight loss in our patients mainly occurred during the first 3 months followed by a plateau and then even a slight regain of weight from the 5th month onwards. This suggests that the preconditioning period could be shortened considerably thereby reducing the risk of severe side effects such as perforation of the gastric wall, nausea, vomiting, and dehydration [17, 26, 28]. In our study population, 6 of 30 patients with gastric balloon experienced considerable side effects. Due to the well-reported risk of severe side effects after gastric balloon insertion, the U.S. Food and Drug Administration (FDA) has issued a warning for some types of gastric balloons [29].

The total weight loss beginning from balloon insertion to 2 years after the operation is comparable with the control group (29.0% vs. 32.2%; *p* = 0.362). There are, however, distinct differences regarding weight loss patterns between both groups which have implications on postoperative patients’ management (e.g., time point of conversion into another surgical procedure) and expectations. Patients with preconditioning lost less body weight during the direct postoperative course compared to controls with an early nadir 12 months after operation followed by weight regain at the time point of 2-year follow-up.

Our findings extend the results of a similar study from of Coffin et al. in several important aspects, including patients after sleeve gastrectomy, etc.) [17]. Most significantly, our follow-up period was 2 years compared to 1 year. This not only revealed weight regain in the pre-treated group but also a complete picture of the body weight dynamic after pre-treatment for the first time.

Our findings are consistent with a preclinical study presenting evidence for a re-programming of a new defended body weight set point after bariatric surgery [30]. One way this could potentially be achieved is through re-sensitization to the adipokine leptin [31]. According to this model, the effects of bariatric surgery on body weight are inversely proportional to circulating leptin levels. Thus, after gastric balloon preconditioning, the presumably lowered leptin levels could be a cause of attenuated weight loss after bariatric surgery.”

Our findings may apply to other conservative preconditioning programs and are important for three reasons. First, they provide a reference for managing patients’ expectations in terms of total weight loss. Fischer et al. showed in their elaborated study that the vast majority of patients overestimate the weight loss achieved by bariatric surgery [32]. There is further evidence that if patients’ expectations are not met this may lead to a poorer overall outcome [33]. Second, the course of weight loss can inform the choice of bariatric procedure. For example, whether to choose one that can be escalated easily (e.g., a three-stage procedure). Third, the early nadir should also be taken into account in order to choose the appropriate time point of escalation. Thus, a reevaluation should be considered 1 year after primary surgery to determine whether a further conversion is needed.

## Conclusion

A multi-stage therapeutic approach with gastric balloon prior to bariatric surgery in super-obese patients does not affect perioperative outcomes. However, while total weight loss among the different groups was similar, the weight dynamics directly after surgery were significantly attenuated after pre-treatment with the gastric balloon. This should be taken into account when choosing the appropriate therapeutic regime and managing patients’ expectations.
